# SerpinB3 as hepatic marker of post-resective shear stress

**DOI:** 10.1007/s13304-023-01531-6

**Published:** 2023-05-19

**Authors:** Enrico Gringeri, Gianmarco Villano, Silvia Brocco, Marina Polacco, Fiorella Calabrese, David Sacerdoti, Umberto Cillo, Patrizia Pontisso

**Affiliations:** 1 Unit of Hepatobiliary Surgery and Liver Transplantation, University of Padova, Via Giustiniani 2, 35128 Padua, Italy; 2 Interdepartmental Center of Experimental Surgery, University of Padova, Via Giustiniani 2, 35128 Padua, Italy; 3 Department of Surgical, Oncological and Gastroenterological Sciences-DISCOG, University of Padova, Via Giustiniani 2, 35128 Padua, Italy; 4 Department of Medicine-DIMED, University of Padova, Via Giustiniani 2, 35128 Padua, Italy; 5 Department of Cardiac, Thoracic, Vascular Sciences and Public Health, University of Padova, Via Giustiniani 2, 35128 Padua, Italy

**Keywords:** SerpinB3, Hepatic marker, Post-resective shear stress, Hepatic resection, Splenectomy

## Abstract

Post-resective liver failure is a frequent complication of liver surgery and it is due to portal hyperperfusion of the remnant liver and to arterial vasoconstriction, as buffer response of the hepatic artery. In this context, splenectomy allows a reduction of portal flow and increases the survival chance in preclinical models. SerpinB3 is over-expressed in the liver in oxidative stress conditions, as a mechanism of cell defense to provide survival by apoptosis inhibition and cell proliferation. In this study, the expression of SerpinB3 was assessed as predictor of liver damage in in vivo models of major hepatic resection with or without splenectomy. Wistar male rats were divided into 4 groups: group A received 30% hepatic resection, group B > 60% resection, group C > 60% resection with splenectomy and group D sham-operated. Before and after surgery liver function tests, echo Doppler ultrasound and gene expression were assessed. Transaminase values and ammonium were significantly higher in groups that underwent major hepatic resection. Echo Doppler ultrasound showed the highest portal flow and resistance of the hepatic artery in the group with > 60% hepatectomy without splenectomy, while the association of splenectomy determined no increase in portal flow and hepatic artery resistance. Only the group of rats without splenectomy showed higher shear-stress conditions, reflected by higher levels of HO-1, Nox1 and of Serpinb3, the latter associated with an increase of IL-6. In conclusion, splenectomy controls inflammation and oxidative damage, preventing the expression of Serpinb3*.* Therefore, SerpinB3 can be considered as a marker of post-resective shear stress.

**Supplementary Information:**

The online version contains supplementary material available at 10.1007/s13304-023-01531-6.

## Introduction

Major hepatic resection (MHR) determines not only parenchymal loss, but also a reduction of intrahepatic vascularization, resulting in an increase of the specific (for gram of tissue) portal flow and of the portal pressure in the remnant liver [1–3]. The shear-stress response favors hepatic regeneration [4]; however, an excessive portal pressure might damage sinusoidal endothelium and Kupffer cells, releasing inflammatory cytokines, with collapse of the microcirculation and hepatocellular injury [5]. In this scenario, the overproduction of reactive oxygen species can contribute to vascular dysfunction and activation of oxidant-sensitive transcription factors [6]. In addition, the hepatic artery buffer response (HABR) generates arterial hypoperfusion of the remnant liver, as consequence of portal hyperperfusion [7]. These features might also be responsible for the biliary ischemic damage and cholestasis [8]. At cellular level, hepatic regeneration is a complex process that involves both mitosis and apoptosis strictly and chronologically regulated in the initial steps. Multiple signaling pathways, induced by inflammation and oxidative stress, lead to the production of growth factors, cytokines, chemokines and molecules which act at the same time in the liver [9–11]. Immediately after hepatectomy, several early genes are activated by transcription factors that are latent in the quiescent liver [12, 13] resulting in increased DNA synthesis, cell size and replication, with preservation of essential metabolic functions [9]. Among cytokines, interleukin-6 (IL-6) is considered a key player, acting not only as mitogenic, but also as an anti-apoptotic factor for hepatocytes, being responsible for the activation of approximately 40% of the involved genes [14, 15].

SerpinB3 (also known as SCCA1, Squamous Cell Carcinoma Antigen) is a protease inhibitor, almost undetectable in normal hepatocytes and over-expressed in hepatocellular carcinoma [16, 17] and in several tumors of epithelial origin [18, 19]. SerpinB3 (SB3) expression has also been demonstrated to increase in response to Tumor Necrosis Factor-α (TNF-α) and Ras-driven inflammation [20, 21], leading to apoptosis resistance [22, 23], cell proliferation and increased IL-6 signaling [24, 25] by acting as an autocrine and/or paracrine mediator. In addition, in transgenic mice, SB3 is associated with a hyperdynamic circulatory syndrome-like pattern, where hepatic artery pulsatility index and portal vein blood flow were significantly increased compared to controls [26]. It has been demonstrated that portal flow diversion can improve the outcome in experimental models of MHR, since rats that underwent splenectomy in association with hepatectomy presented levels of transaminases significantly lower than those with hepatectomy alone [27]. Furthermore, splenectomy associated to extreme liver resection can balance the HABR mechanism, since a greater flow of the hepatic artery has been described in rats with major hepatectomy and splenectomy compared with those without splenectomy [28, 29].

In this study, we have assessed the possible role of SB3 as a marker of shear stress using an in vivo rat model of MHR in the presence or absence of splenectomy.

## Methods

### Experimental model

Twenty-one male Wistar rats, weighting 220 to 390 g, were housed for at least 7 days in a light- and temperature-controlled room with access to food and water. Rats, randomly divided into 4 groups, underwent the following procedures:

- Group A: 30% liver resection (N. 5 rats),
- Group B: > 60% liver resection (N. 7 rats),
- Group C: > 60% liver resection and splenectomy (N. 6 rats),
- Group D: sham operated, control group (N. 3 rats).

The animals were bred at the Animal Care Facility of the Experimental Surgery Division of the University of Padova. Surgical operations were performed under the Italian law (n° 116/92), in accordance with European Union regulations. All procedures, including echo Doppler ultrasound, blood and tissue sampling have been performed under general anesthesia induced by the inhalatory administration of Sevoflurane (4 ml/min for the induction and 1.5–3.5 ml/min for the maintenance, according to reduced hepatic metabolization due to different size liver resections) that was mixed to oxygen 0.5 L/min throughout the procedure.

Immediately before and 2 h after the surgical procedures, blood samples were obtained from the caudal vein of the rats from each group to assess standard liver function parameters including aspartate aminotransferase (AST), alanine aminotransferase (ALT), lactate dehydrogenase (LDH), Ammonium and Lactic Acid.

After midline laparotomy, hepatic and splenic intra-operatory echo Doppler ultrasound was carried out in each group. Liver lobules were subsequently mobilized by cutting the falciform and hepatogastric ligaments. The liver mass was reduced by resection of the left lateral lobe (Group A) and the left lateral, right lobe and caudate lobe (Group B and C). Liver resection was carried out with piercing sutures through the liver parenchyma with 5/0 silk. Rats from Group C received splenectomy together with liver resection and the splenic vessels were sectioned at the hilum after sutures with 5/0 silk. The control Group D did not receive any resection. The resected liver parenchyma was weighted and stored for further analysis, while the abdomen was closed by a suture in layers.

After an observation period of 2 h all the animals were subjected to blood sampling, as previously described, re-laparotomy and intra-operatory echo Doppler ultrasound. The remnant liver underwent en-bloc hepatectomy, was weighted and frozen in part, while the remaining liver specimens were fixed in 10% buffered formaldehyde and embedded in paraffin. Animals were killed by the inhalatory administration of CO_2_.

### Echo Doppler ultrasound measurements

Echo Doppler ultrasound of splanchnic vessels was carried out in each group using a dedicated apparatus (Vevo2100 Visualsonics, probe MS-550D, 22–55 MHz) equipped with heated table that allowed monitoring of heart frequency, respiratory frequency and electro-cardiography as previously reported [30]. To obtain an optimal ultrasound interface, peritoneal cavity of each rat was filled with heated sterile saline solution, to permit adequate Doppler measurements without compressing examined parenchymas. Portal vein (PV) diameter, velocity and flow were assessed immediately upstream the emergency of the first portal branch. The Hepatic Artery (HA), Pulsatility Index (PI) and Resistance Index (RI) were evaluated at the hepatic hilum, while the Splenic Artery (SA), PI and RI were measured at the splenic hilum.

### Histopathological analysis

Standard staining of the remnant liver with hematoxylin–eosin was used for light microscopy and the histopathological parameters analyzed were: vascular congestion, edema and glycogenic charge.

### Molecular techniques

The frozen liver specimens were analyzed by real-time PCR to assess mRNA expression of Sb3, genes linked to inflammation as IL-6, TNF-α and oxidative stress genes as Heme Oxygenase 1 (HO-1), NADPH Oxidase 1 (Nox1) and NADPH Oxidase 2 (Nox2).

Total RNA was extracted using RNeasy Mini Kit (Qiagen GmbH, Hilden, Germany) according to the manufacturer’s instructions. After determination of the purity and the integrity of total RNA, 2 µg of each sample was reverse transcribed in cDNA using iScript cDNA Synthesis Kit (Bio-Rad, Milan, Italy). The reaction was performed at 25 °C for 5 min, then activation of the reverse transcriptase at 42 °C for 30 min and lastly at 85 °C for 5 min.

The real-time PCR was performed by the SYBR Green assay using a FastStart DNA MasterPLUS SYBR Green KitTM (Roche, Monza, Italy) in glass capillaries where the specimen was mixed with a blend containing a fluorescent molecule. After an initial denaturation step at 95 °C for 10 min, 45 cycles of amplification were carried out and included the following conditions: denaturation at 95 °C for 10 s, annealing at 60 °C for 30 s and extension at 72 °C for 10 s. Amplification of specific transcripts was confirmed by melting curve profiles at the end of each PCR cycle, using the specific routine built-up in the Light Cycler instrument. The housekeeping gene Hypoxanthine Phosphoribosyl Transferase (HPRT) was amplified in parallel in all amplification sets.mRNA expression of interest genes was calculated according to the threshold cycle of individual genes and the results were expressed as a relative ratio of the target to the housekeeping gene using the Light Cycler Relative Quantification software 4.05 (Roche Diagnostics, Monza, Italy). A negative control, samples containing all reagents but no cDNA template, were included in all runs.

Primers, designed from sequences of rat derived from the GenBank database using Primer 3 (Whitehead Institute, Massachusetts, USA) and Operon’s Oligo software (Operon, California, USA) and purchased from Eurofins MWG (Ebersberg, Germany) are reported in Table 1 (Supplementary material).

### Statistical analysis

Each basal biochemical value, gene expression value and echo Doppler parameter has been compared to the basal values of the other groups and the same was carried out for the values obtained after the observation time. Moreover, a comparison was made between the basal and the final values in the context of each group. The following non-parametric tests have been chosen: Wilcoxon test for the comparison between two groups of paired data, Kruskal–Wallis test and Dunn post test for the comparison among more than two groups and Spearman test for the correlation in gene expression. Significance was established at *p* value < 0.05. The statistical significance of all tests used in the study was Employed software: GraphPad InStat (San Diego, CA, USA).

## Results

### Biochemical parameters

The group of rats that underwent > 60% hepatectomy showed a higher increase of biochemical parameters, including transaminases (AST, *p = *0.017; ALT, *p = *0.017) and ammonium (*p = *0.048), compared to the groups of sham-operated controls and rats with > 60% hepatectomy and splenectomy, where the ammonium levels were even below the median values observed in controls. Among the groups with > 60% liver removal, in rats with associated splenectomy, transaminases showed a higher, although not significant trend increase, possibly due to the major surgical time of the procedure.

**Fig. 1:**
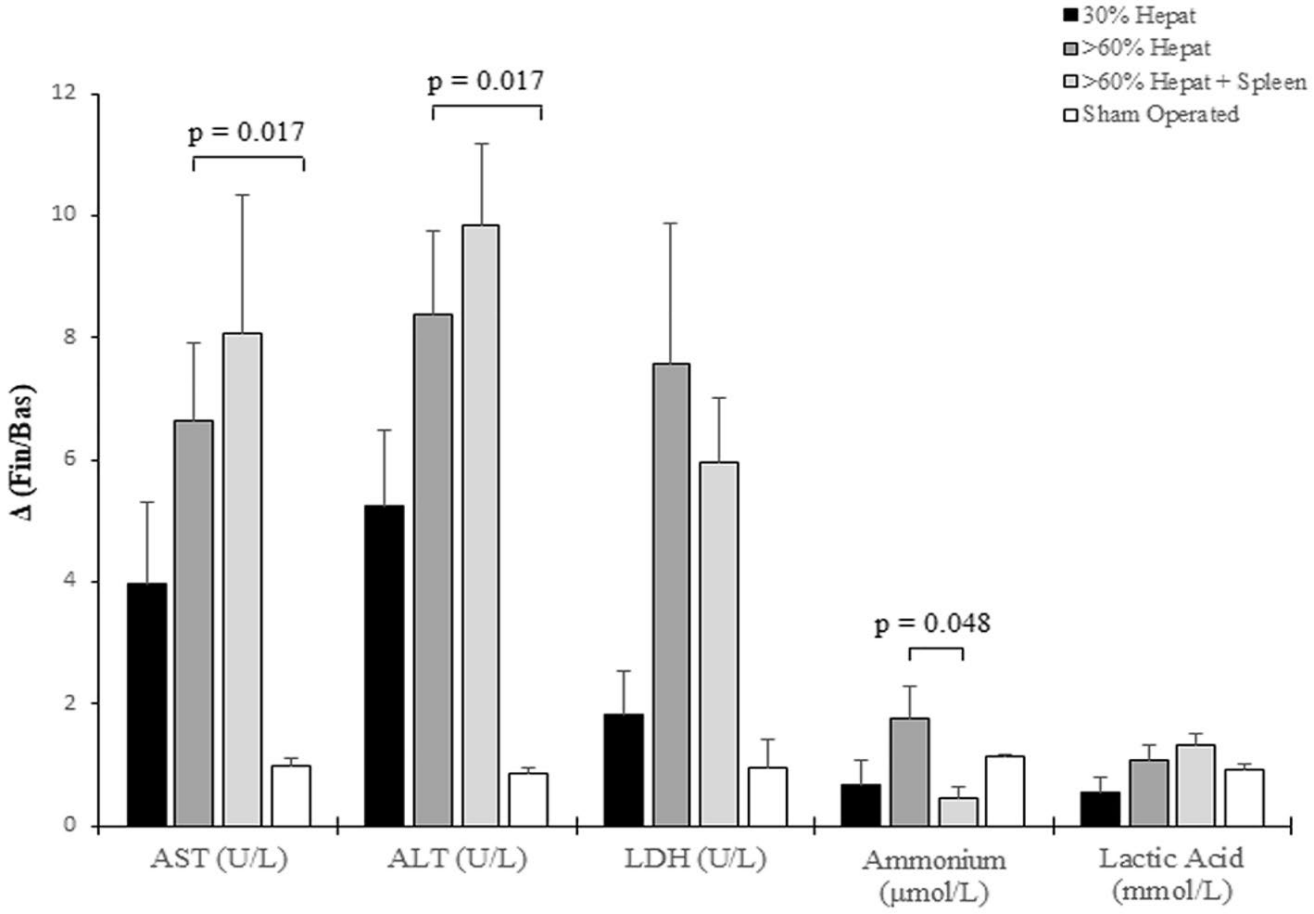
Biochemical parameters in rats that underwent different degrees of hepatectomy. Columns represent median values and vertical bars represent standard error median. The biochemical parameters were expressed as fold increase relative to basal values, *p < *0.05

In addition, no significant differences between pre-operative and post-operative values were observed for LDH, tended to be more elevated in the group with major hepatectomy, and Lactic Acid in each group and neither among different groups (Fig. 1).

### Hemodynamic effects

#### Portal vein flow velocity

**Fig. 2:**
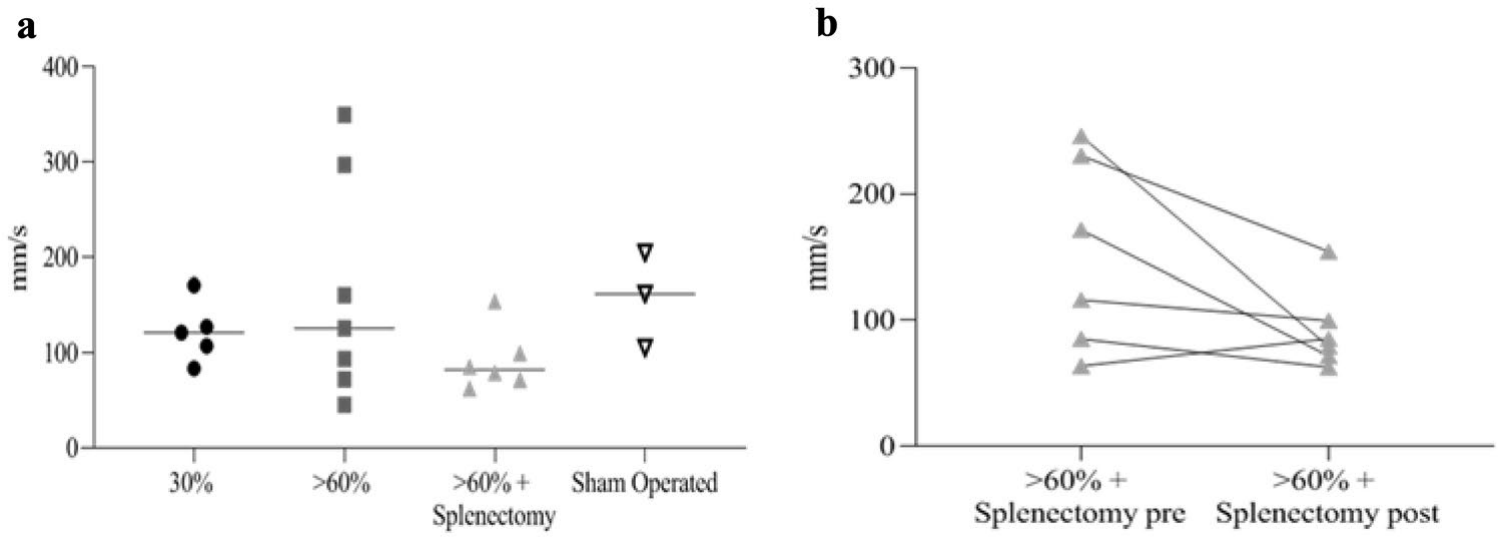
Echo Doppler ultrasound results in PV. **a** Mean PV velocity after surgery, *p = *0.213 (ns). **b** Mean PV velocity pre and post > 60% hepatectomy and splenectomy, *p = *0.094 (ns)

**Fig. 3:**
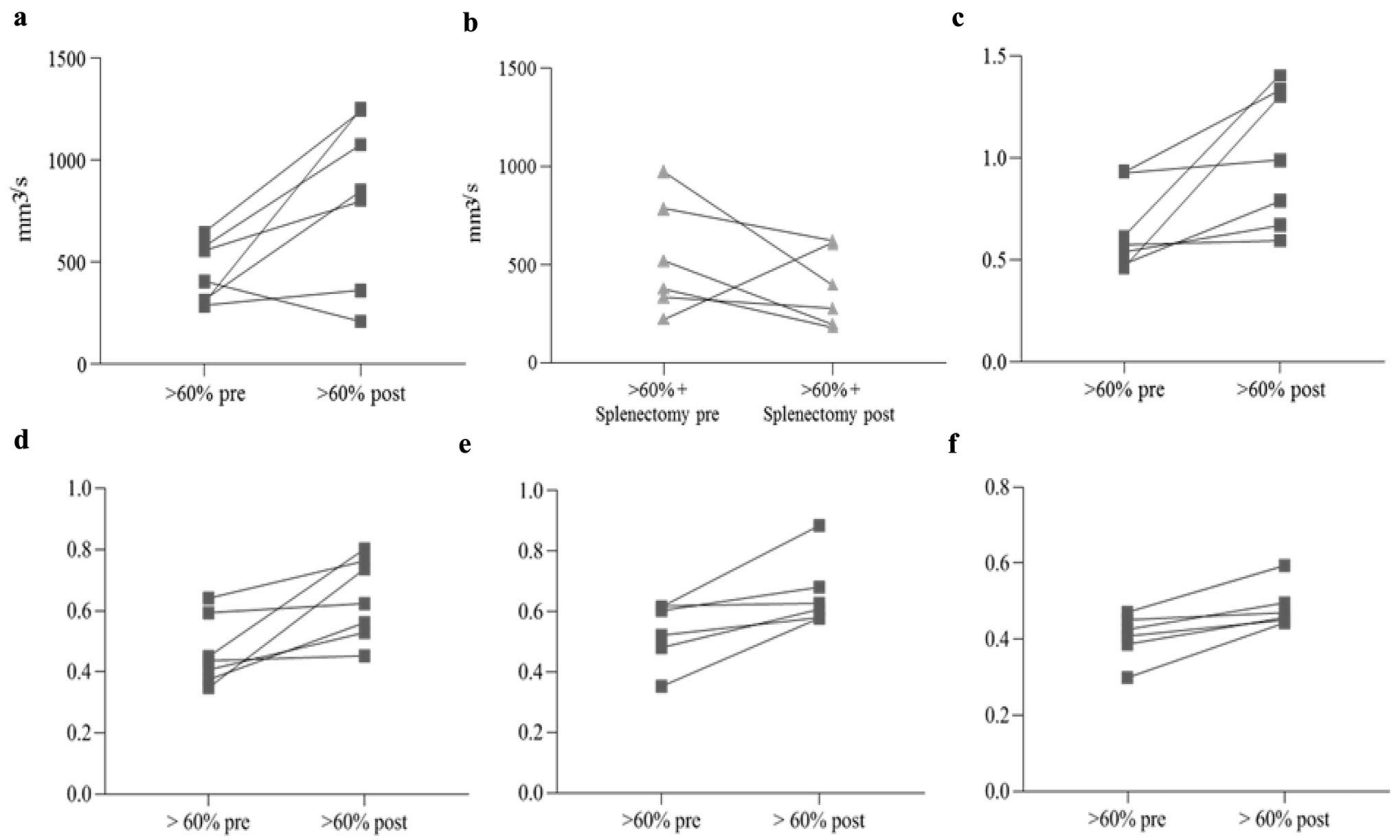
Echo Doppler ultrasound results in PV, HA and SA. **a** Mean PV velocity pre and post > 60% hepatectomy and splenectomy, *p = *0.047. **b** PV flow pre and post > 60% hepatectomy, *p = *0.047. **c** HA Pulsatility Index (HA-PI) pre and post > 60% hepatectomy, *p = *0.016. **d** HA Resistive Index (HA-RI) pre and post > 60% hepatectomy, *p = *0.016. **e** SA Pulsatility Index (SA-PI) pre and post > 60% hepatectomy, *p = *0.031. **f** SA Resistive Index (SA-RI) pre and post > 60% hepatectomy, *p = *0.031

Overall mean portal velocity in each group did not show significant variations after surgical intervention (Fig. 2a), although the comparison between pre- and post-operative median velocities in group with splenectomy showed a trend in reduction even if it did not achieve statistical significance (Fig. 2b). These findings were not associated with changes of PV diameter. The median values of right portal flow showed a significant increase in post-operative period in the group of rats with > 60% hepatectomy (*p = *0.047) (Fig. 3b). No statistical difference was evident among the other groups, even if in rats with > 60% hepatectomy with splenectomy a trend in reduction of portal flow was observed in post-operative period, likely due to removal of spleen contribution to portal flow (Fig. 3a and b).

### HA Doppler measurements

HA hemodynamic evaluation was carried out analyzing Pulsatility Index (HA-PI) and Resistive Index (HA-RI). Considering the median PI and RI of HA, we observed a significant increase between the pre-operative and post-operative period in the group of rats with > 60% hepatectomy (*p = *0.016 for both HA-PI and HA-RI) (Fig. 3c and d), while pre- and post-operative values were similar in the other groups.

### SA Doppler measurements

As for HA, even for SA Pulsatility Index (SA-PI) and Resistive Index (SA-RI) were considered. The median PI and RI of splenic artery could not be measured in one animal of group B due to technical reasons and in rats of group C in post-operative period due to splenectomy.

As shown in Fig. 3e and 3f, both PI and RI showed a significant increase in post-operative period in group with > 60% hepatectomy (*p = *0.031 for both), while these parameters were unchanged in the group of rats with 30% hepatectomy and in sham-operated controls.

### Liver tissue analysis

**Fig. 4:**
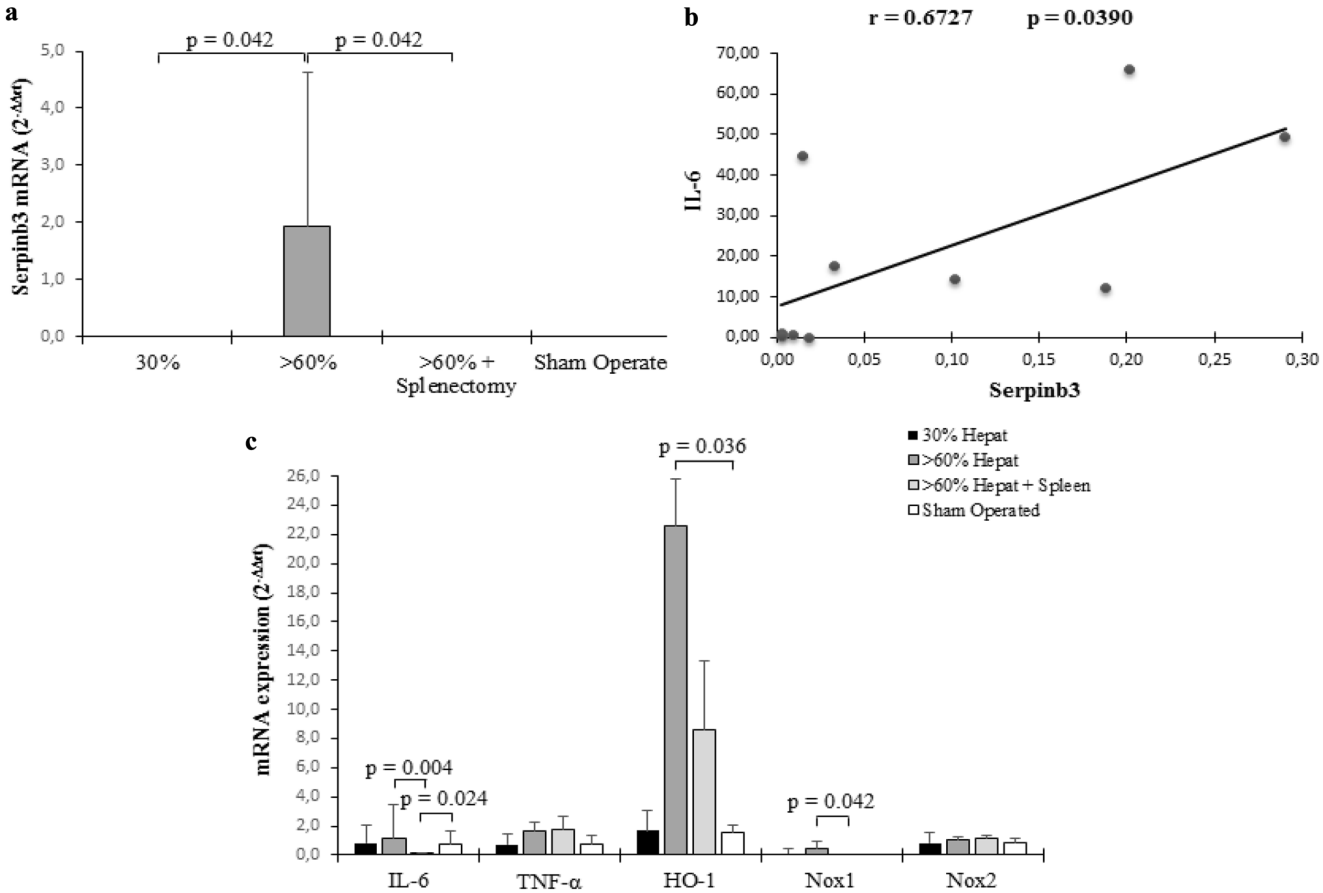
mRNA expression in rat liver tissue detected by real-time polymerase chain reaction. **a** mRNA expression of Sb3 in rat liver after different degrees of hepatectomy. **b** Spearman rank correlation analysis of Sb3 mRNA and IL-6 mRNA levels. **c** mRNA expression of inflammatory cytokines and oxidative stress genes. Changes in mRNA gene expression are expressed as fold increase relative to resection at time 0 using the 2^−ΔΔCT^ method. The results were normalized to the HPRT housekeeping gene. Columns represent median values and vertical bars represent standard error median

A rise in Sb3 levels, that is not physiologically detectable in normal liver, was observed after 2 h from surgery in the remnant liver only in rats that underwent > 60% hepatectomy (*p = *0.042), while remained undetectable in the livers of all the other groups of rats, including those with > 60% hepatectomy with associated splenectomy (Fig. 4a).

In parallel with these findings, only in the remnant liver of rats with > 60% hepatectomy a significantly higher upregulation of IL-6 (*p = *0.004), of HO-1 (*p = *0.036) and of Nox1 (*p = *0.042) was observed, while these molecules were not upregulated in the group of rats with > 60% hepatectomy and splenectomy. It is worth to note that a significant overall direct correlation between IL-6 and Sb3 expression levels (Spearman test r = 0.6727, *p = *0.039) was observed (Fig. 4b). TNF-α and Nox2 were not significantly affected by surgical procedures (Fig. 4c), at least at the chosen times of analysis.

### Histopathologic analysis

In the remnant liver of the groups of rats with > 60% hepatectomy, either with or without splenectomy, a reduction of glycogen amount and mild edema increase was observed 2 h after resection, while vascular congestion was more increased in the group without splenectomy than in that with splenectomy (Fig. 5, Supplementary material), supporting the higher shear stress of the former group.

## Discussion

The present study was carried out to evaluate the effect of different approaches of extreme hepatectomy on shear stress and to assess the possible usefulness of SB3 expression to monitor this phenomenon. Extreme hepatectomy (> 60%) with or without splenectomy determined a significant cytolysis with increased transaminase release, as previously described [29]. However, porto-arterial hemodynamics revealed a decreased relative portal flow and HABR response in rats that underwent splenectomy in association with extreme hepatectomy, supporting other preclinical studies, where different types of measurement were used [28].

At the same time, massive hepatectomy induced an increase in hepatic PI and RI, indicating arterial vasoconstriction that was not observed in minor hepatic resections or in case of associated splenectomy. Thus, the association between high-volume hepatectomy and splenectomy, showing mean portal flow and hepatic arterial resistance similar to minor hepatic resection and control groups, seems to prevent the hepatic hemodynamic alterations caused by excessive portal flow and associated HABR. The reduction of portal flow, determined by the splenectomy associated with MHR, has been indeed found to improve survival and to decrease hepatic cell damage [27, 28]. On the other hand, in rats with high-volume hepatectomy without splenectomy an increase in splenic arterial resistive and pulsatility index was registered, that can reflect the occurrence of some degree of portal hypertension determined in this case, as previously shown in humans [31].

As result of hemodynamic alterations following liver resection, a wide number of homeostasis regulators, as transcriptional factors, growth factors and cytokines are induced [32], leading to increased oxidative stress and hypoxia, likely accelerating liver regeneration [33]. In particular, oxidative stress conditions can induce the synthesis and release of SB3 [23], a molecule that we have previously observed to confer, after partial hepatectomy, resistance to apoptotic cell death and an additional stimulus for liver cell proliferation, leading to a final improvement of liver growth, at least in part determined by IL-6 production [24].

On basis of the scientific evidences about the activity of SB3 [19], this molecule might counteract acute liver damage by activation of pathways leading to hepatic regeneration, apoptosis inhibition and oxidative stress reduction. In fact, the levels of Sb3 raised only after MHR, where the shear stress was higher than in the other groups of animals, exposed to lower hepatectomy or protected by splenectomy. In agreement with these findings, the remnant livers of rats that underwent MHR showed not only higher values of the oxidative stress molecule HO-1, as shown by Solangi et al. [34], but also increased levels of IL-6, that were positively correlated with Sb3, likely acting as protective molecular response in this contest. IL-6 acts on hepatocytes via the IL-6 receptor, activating the signal transducer and activator of transcription 3 (STAT3) that in turn activates transcription of target genes at nuclear level [35–37] leading to the initiation of liver regeneration [38–40]. It is worth to note that STAT3 is also able to bind the promoter of SB3 activating its expression [41] and determining a positive loop between these two molecules.

In conclusion, in our experimental context of MHR, the resulting increased shear stress due to portal hyperperfusion, associated with increased arterial resistance, determines increased liver oxidative stress that up-regulates the expression of Sb3, likely as a protective mechanism. Therefore, SB3 in the remnant liver might be considered as a hepatic marker of post-resective shear stress. If these results will be confirmed in surgical practice, this molecule could become a prognostic parameter that could support clinical decisions.

## Supplementary Information

Below is the link to the electronic supplementary material.

Supplementary file1 (DOCX 307 kb)

## Data Availability

The data presented in this study are available on request from the corresponding author.
