# Supplementary material for: SerpinB3 as hepatic marker of post-resective shear stress
Source: Updates Surg. 2023 May 19;75(6):1541–8. doi: 10.1007/s13304-023-01531-6 (PMC10435418; doi:10.1007/s13304-023-01531-6)
Supplement: Supplementary file 1 — Supplementary file1 (DOCX 307 kb) [file 13304_2023_1531_MOESM1_ESM.docx]

**Supplementary material**

| **Gene**  **name** | **Forward primer sequence (5'-3')** | **Reverse primer sequence (5'-3')** |
| --- | --- | --- |
| **Serpinb3** | ATGGTCGATGCTTTCAATCC | TGTGGTCCTTGGTGCAGATA |
| **IL-6** | AAGCCAGAGTCATTCAGAGCAA | GGTCCTTAGCCACTCCTTCT |
| **TNF-α** | ATGGGCTCCCTCTCATCAGT | GCTTGGTGGTTTGCTACGAC |
| **HO-1** | AGAGTCCCTCACAGACAGAGT | TAAATTCCCACTGCCACGGTC |
| **Nox1** | GAGTGAAAGTCATCCCCGCA | TAAAAAGCAATCGGCGCGAG |
| **Nox2** | TTGCCGGAAACCCTCCTATG | CGGGACGCTTGACGAAAATG |
| **HPRT** | GGTCCATTCCTATGACTGTAGATT | CAATCAAGACGTTCTTTCCAGTT |

**Table 1** Nucleotide sequences of the rat gene primers used in the study


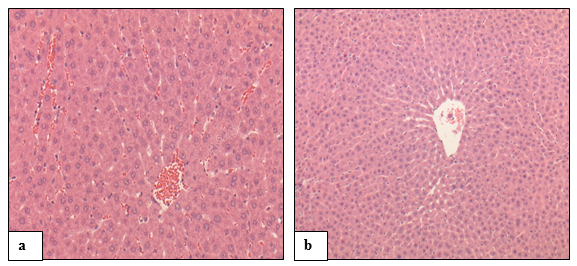


**Fig. 5** Example of the degree of oedema and vascular congestion in the remnant liver of >60% hepatectomy (**a**) and of >60% hepatectomy with splenectomy (**b**) in rat model. In >60% hepatectomy group hematoxylin eosin shows grade 1 (10%) oedema and grade 2 (40%) vascular congestion. In >60% hepatectomy with splenectomy group hematoxylin eosin shows grade 1 (10%) oedema and grade 1 (30%) vascular congestion. Original magnification 20X
